# AIKYATAN: mapping distal regulatory elements using convolutional learning on GPU

**DOI:** 10.1186/s12859-019-3049-1

**Published:** 2019-10-07

**Authors:** Chih-Hao Fang, Nawanol Theera-Ampornpunt, Michael A. Roth, Ananth Grama, Somali Chaterji

**Affiliations:** 1 Department of Ag. and Biological Engineering, Purdue University, West Lafayette, IN USA; 2 College of Computing, Prince of Songkla University, Bangkok, Thailand; 3 Google Inc., Mountain View, California USA; 4 Department of Ag. and Biological Engineering, Purdue University, Purdue University, IN USA

**Keywords:** Support vector machines (SVM), Random forest (RF), Deep neural networks (DNN), ConvNets (CNN), Image processing algorithms, Distal regulatory elements, Enhancers, Silencers, Epigenomics, ROC curves, Graphics processing units (GPU), Hyperparameter tuning, NIH roadmap epigenomics

## Abstract

**Background:**

The data deluge can leverage sophisticated ML techniques for functionally annotating the regulatory non-coding genome. The challenge lies in selecting the appropriate classifier for the specific functional annotation problem, within the bounds of the hardware constraints and the model’s complexity. In our system Aikyatan, we annotate distal epigenomic regulatory sites, e.g., enhancers. Specifically, we develop a binary classifier that classifies genome sequences as distal regulatory regions or not, given their histone modifications’ combinatorial signatures. This problem is challenging because the regulatory regions are distal to the genes, with diverse signatures across classes (e.g., enhancers and insulators) and even within each class (e.g., different enhancer sub-classes).

**Results:**

We develop a suite of ML models, under the banner Aikyatan, including SVM models, random forest variants, and deep learning architectures, for distal regulatory element (DRE) detection. We demonstrate, with strong empirical evidence, deep learning approaches have a computational advantage. Plus, convolutional neural networks (CNN) provide the best-in-class accuracy, superior to the vanilla variant. With the human embryonic cell line H1, CNN achieves an accuracy of 97.9% and an order of magnitude lower runtime than the kernel SVM. Running on a GPU, the training time is sped up 21x and 30x (over CPU) for DNN and CNN, respectively. Finally, our CNN model enjoys superior prediction performance vis-‘a-vis the competition. Specifically, Aikyatan-CNN achieved 40% higher validation rate versus CSIANN and the same accuracy as RFECS.

**Conclusions:**

Our exhaustive experiments using an array of ML tools validate the need for a model that is not only expressive but can scale with increasing data volumes and diversity. In addition, a subset of these datasets have image-like properties and benefit from spatial pooling of features. Our Aikyatan suite leverages diverse epigenomic datasets that can then be modeled using CNNs with optimized activation and pooling functions. The goal is to capture the salient features of the integrated epigenomic datasets for deciphering the distal (non-coding) regulatory elements, which have been found to be associated with functional variants. Our source code will be made publicly available at: https://bitbucket.org/cellsandmachines/aikyatan.

**Electronic supplementary material:**

The online version of this article (10.1186/s12859-019-3049-1) contains supplementary material, which is available to authorized users.

## Background

Eukaryotic chromosomes comprise of mosaics of accessible (euchromatin) and inaccessible (heterochromatin) domains whose regulation is controlled by regulatory elements such as promoters, enhancers, and silencers. Further, it is estimated that the human genome contains approximately 20,000 to 25,000 genes representing only 2% of the genomic sequence, while 98% of the genome is non-coding. The non-coding genome includes maintenance elements (e.g., centromeres and telomeres) and origins of replication that control DNA repair and replication processes; regulatory elements such as promoters, enhancers, silencers, insulators; and regulatory RNAs (micro-RNAs), which regulate the spatial, temporal, and cell-type specific expression of genes. Thus, transcriptional regulation of genes is a complex orchestration, subject to DNA folding mechanisms and feedback regulatory controls. The regulatory controls are accomplished not only by proximal promoters, but also by distal regulatory elements, such as, enhancers, superenhancers or stretch enhancers, insulators, and silencers [1]. Promoters initiate the transcription process at the transcription start site (TSS), mediated by transcription factors (TFs) and other chromatin-modifying enzymes. Enhancers upregulate gene expression in a distance- and orientation-independent manner. They do so by displaying binding sites for ubiquitous and cell-specific TFs and “looping” to get situated closer to the genes that they target for regulation at that point of space and time [2]. Thus, enhancers can be separated from the promoters that they regulate by thousands of base pairs, often situated on different chromosomes, and are drawn close to the transcription factories or active chromatin hubs during gene activation. Further, there are insulators that can restrict the long-range regulation of genomic enhancers and silencers (barriers), conceptualized as specialized derivatives of promoters [3], and potentially acting in either capacity, as dictated by the biological process [4]. The fact that these distal regulatory elements (DREs) lack common sequence features and often reside far away from their target genes has made them difficult to identify. Further, the annotation of the non-coding genome is an active research area, with discoveries in epigenomic regulatory elements uncovering functional features of DNA (epigenomic marks such as histone modifications, DNA methylation, and genome folding) associated with gene regulatory domains, in myriad cell types and organisms [5–7].

In AIKYATAN, we solve the problem of predicting distal regulatory elements from the DNA sequences, captured by histone modifications, in the vicinity of p300 co-activator binding sites in the DNA.

We wish to annotate distal regulatory elements (DREs)—located distal, in a two-dimensional sense, to the genes that they regulate—comprising of enhancers, insulators, locus-control regions, and silencing elements. While the last decade has seen rapid progress in the development of experimental techniques to identify these regulatory elements on a genome-wide scale, the characterization of the epigenomic features that confer regulatory power to these regions is limited [8–10]. Of these studies, the focus has primarily been on enhancers, and to some extent, on insulators, which contribute to cell-type specific gene expression in distinct ways. Thus, we wish to increase the scope of predictive algorithms to extensively annotate the varied types of long-range regulatory elements, “learning” their combinatorial histone modification signatures. This superset can then be pipelined into a more specific classifier, such as one for identifying enhancers, e.g., EP-DNN [11], to tease out genomic enhancers from this superset of DREs. Further, the residual DREs can then be clustered into other kinds of long-range regulators by unraveling their unique signatures using unsupervised learning or interpretable algorithms, such as [12]. Interpretable algorithms, in this problem, can be advantageous because interpretability will result in possible listing of feature-importance scores for different histone modifications and TFs that result in precise and computationally efficient predictions for target DREs. This can enable the identification of newer types of DREs, given that the preprocessing step would decrease some of the noise in the data sets that we started with. Many types of ML techniques have been applied for classification problems in epigenomics, where the data has the characteristics of being both noisy [13] and multi-dimensional [14, 15]. **We build a fast and accurate classifier for answering the binary question of whether a genomic sequence is a distal regulatory element or not, while taking into consideration the following criteria when building our classifier.**

- **Computational complexity of the ML model:** The chosen ML model should be able to process high data volumes with a large number of training examples (*n*), with the additional constraint of inpterpolating for incompleteness and interpreting high-dimensional features (*d*), the often cited curse of dimensionality, which is ingrained in (epi)genomic data sets. Otherwise, one has to use either feature selection or dimensionality reduction on the original input space in order to reduce *d*, using a method similar to [12], or sub-sampling the training set for learning, potentially obfuscating the real data distribution. For example, the distribution of genomic data sets is often found to be skewed normal due to the fact that there may be a small class of genes that demonstrate a high level of connectivity in biological networks forming “network hubs” [16], while the more ubiquitous specialized genes control a smaller subset of biological processes, forming smaller networks and participating in fewer of those as well.
- **Learning the structure of the data:** The chosen ML model should be able to extract knowledge from the structure of the data, which in this domain has a three-dimensional contour offering a complexity similar to that encountered in computer-vision problems. Otherwise, more often than not, a lower-complexity model may introduce unacceptable bias in the learning. We find this empirically for our linear SVM variant of Aikyatan, which is mitigated through the use of the kernel variant, as we have seen in other problems in the epigenomic annotation space [17, 18]. In the same vein, a simple ANN-based model when converted to a deeper model resulted in a 12% increase in our prediction accuracy in a related epigenomics classification problem that we solved recently, classifying genomic sequences as targets of non-coding regulatory RNA [17]. Thus, in most cases, we find that with some loss in interpretability, a non-linear model can handle epigenomic datasets more accurately [19–21].

Among all types of classifiers, Support Vector Machines (SVM) are robust inferencing machines requiring minimal parameter choices that can be generalized into higher-dimensional spaces using kernel methods. If the data in the input space is linearly separable, then a linear SVM guarantees perfect separation, else a non-linear kernel, such as a Radial Basis Function (RBF) kernel, SVM is recommended. Another approach to increase the prediction performance is to use ensemble methods. Random forest is a popular method in this category and has been proven to be useful for prevent overfitting. [22]. However, the memory and the inference time grows as a function of number of training samples [23], preventing random forest from being widely used in large-scale data analysis. Looking at the large volumes of data available in our problem domain, plus the additional high-dimensionality attribute [20], neural networks coupled with GPU backends, felt like the natural alternative. With this in mind, we consider both vanilla Deep Neural Networks (DNN) and Convolutional Neural Networks (CNN) in this work. In recent years, CNNs [21–24] have demonstrated success in computer vision, especially in image-classification and recognition tasks. The computer vision applications of CNNs stem from the design of CNNs being highly correlated to the structure of images and their ability to pool the parameters of the image using kernels or filters resulting in data-compression, and to some extent, denoising of the images. Our input can be regarded as snapshots of chromatin signals at specific DNA locations, encoding the spatial notion of the genome into our learning, which enables a CNN to extract distinctive features locally. Thus, a CNN is hypothesized to be effective in extracting image-like input features from the two-dimensional combinatorial histone modification signatures’ data.

With the above discussion as a guiding principle, we selected a suite of ML protocols under the banner AIKYATAN^1^, e.g., linear SVM, kernel SVM, random forest, DNN, and CNN, specifically targeted to this problem and using chromatin-based features, namely, 24 histone modifications’ signatures as feature set, for the classification task.

We train our models on p300 co-activator binding sites; H1-specific, transcription factor binding sites (TFBS): NANOG, OCT4, and SOX2; and uncondensed, cleavage-sensitive, DNase I Hypersensitivity Regions (DHS); which are all distal to TSS; as positive examples. The TSS sites and random genomic regions, which are known to be distal to DHS, are treated as negative samples. Our empirical results show that linear SVM, while being computationally tractable, gives poor accuracy, indicating the bias in the linear model underfitting to the non-linearity embedded in the data. Random forest achieves similar performance compared to linear SVM but still could not outperform kernel SVM and DNN-based models with increasing training set sizes. Kernel SVM significantly improves the accuracy but slows down significantly to the point where it is not feasible to use for a significant-sized genomic dataset. *For example, in our case, with a 1 GB training set size (226k samples), kernel SVM takes ≈ 4.5 days to compute. Consider that by comparison the total dataset for the histone modifications signatures’ feature file alone, after data preprocessing, for the human embryonic stem cell line H1, as available from the NIH Roadmap Epigenomics Mapping Consortium [*52], is 133 GB (30M samples). Further, the computational cost when performing parameter tuning through cross-validation is multiplied by the number of parameter combinations. A DNN achieves comparable accuracy to a kernel SVM, albeit, with a higher training set size, which is readily available in our problem domain. *As a strong point for deep learning algorithms, their training time grows linearly with training set size while the training time for kernel SVM is between a quadratic and cubic function of the number of training samples. In addition, the testing time for kernel SVM grows linearly with number of support vectors.* Quantitatively, we find that on a CPU backend, the DNN model is 29.x faster than kernel SVM. Finally, in AIKYATAN, we find that CNN is best-in-class and we call it **DRP-CNN** (**D**istal **R**egulatory Site **P**rediction using **C**onvolutional **N**eural **N**etworks). We find that CNN performs better than DNN in terms of prediction accuracy. With the largest training set of 16 GB (3.6M samples), CNN achieves 2% higher than DNN for the Validation-Rate experiment, even though for the latter, the DNN model had already reached a 95.8% prediction accuracy. In addition, our empirical results also show that **DRP-CNN** enjoys superior prediction performance vis-à-vis the state-of-the-art methods most pertinent to our problem, e.g., CSIANN [30] and RFECS [31]. Note that these methods solve a simpler task of enhancer prediction while AIKYATAN predicts all distal regulatory elements.

To further reduce the computational burden of training, we use Graphics Processing Units (GPUs) for our task, as they can significantly reduce the running time required by CPU-based implementations due to their higher memory bandwidth and computational capability. This allows for more elaborate parameter optimization, critical to the success of deep learning models. For our experiments, we use Keras [26] as the frontend and Theano [27] as the backend. With GPU enabled, the training time is sped up by 21x and 30x over a CPU, for DNN and CNN, respectively.

Main Contributions:

1. We motivate the use of deep learning variants for our problem of predicting which genomic sequences represent DREs and show how to build an ML classifier based on a Convolutional Neural Network (RP-CNN) for this biologically important use case. Specifically, we demonstrate how we formulate histone modification signals as snapshots and demonstrate how local feature extraction and the shift-invariant property of a CNN can apply to histone modification signals and combinatorial functions. This illustrates the applicability of CNNs to biological data that is distinct from typical image datasets. Our empirical results support our hypothesis and show that CNN is the best model to capture these epigenomic patterns, achieving a validation rate of 97.9% or higher. An added benefit is the local connectivity and parameter sharing property of CNNs resulting in dramatic reduction in the number of parameters, while also contributing to the shift invariance property. This is important in the genomics context given the steep rise in the volume and variety of datasets.
2. We show that a linear SVM and random forest are not expressive enough for the classification task and a kernel SVM, although theoretically powerful, cannot achieve as high a test accuracy as deep learning approaches due to its high training time complexity. This attribute limits the kernel SVM model from learning all the underlying patterns and nuances present in the entire data set, with this complexity increasing with the perpetual increase in data volumes and varieties in genomics, rightly described as a four-headed beast because of the complexity in data acquisition, storage, distribution, and analysis [30].
3. We show how a GPU-backend for the deep learning task speeds up the training process and makes it feasible to deploy our algorithmic variants for high-throughput processing on a large-sized, biologically relevant data set. In addition, we give comprehensive empirical results on the comparison of both training and testing times for Aikyatan as well as state-of-the-art methods, such as RFECS [31], and CSIANN [30], on CPUs.

### Related Works

In this subsection, we discuss recent works on predicting a specific set of distal regulatory elements, enhancers, which is the closest classification task to our problem at hand. We select two leading-edge ML models that are representative of two different classes of ML algorithms, random forest (RFECS) and artificial neural networks with Fisher discriminant analysis (CSIANN), respectively for detailed benchmarking of AIKYATAN. However, it is important to note that AIKYATAN solves the larger classification problem of whether or not genomic sequences are distal regulatory elements or not, rather than the classification of genomic enhancers that all of these competitors solve. Of these, enhancers are the most versatile in their usage in the genome and by current estimates, there are over a million enhancers in the genome. We believe solving the overall distal regulatory element problem is useful because it is allows us to classify (downstream) different classes of distal regulatory elements rather than annotating genomic enhancers alone.

RFECS (Random forest-based Enhancer identification from Chromatin States) [31] proposed a random forest algorithm to predict p300 enhancers from combinatorial patterns of histone modifications in H1 and IMR90 cell types. CSIANN [30] used Fisher Discriminant Analysis for Feature Extraction/Reduction and use 1 hidden layer Time-delay neural network for Enhancer Prediction. DEEP [32] utilized a framework of an ensemble of kernel SVMs with an overarching artificial neural network (ANN) to predict enhancers in different cell types using features derived from histone modifications and sequence characteristics. More recently, EP-DNN [11] used a DNN, demonstrating superior accuracy of 91.6%, relative to 85.3% for DEEP-ENCODE and 85.5% for RFECS. PEDLA (predicting enhancers with a deep learning-based algorithmic framework) [33] implemented a Hidden Markov Model (HMM) with DNN-based probabilities to predict enhancers from distinct categories of heterogeneous data, including histone modifications, TFs’ and co-activators’ binding, DNA methylation, sequence characteristics, etc., and achieved superior performance compared to the previous approaches. PEDLA’s key novelty comes from the integration of diverse datasets rather than the process of extracting maximum signal from a single combinatorial epigenomic dataset (e.g., histone modifications’ signatures for AIKYATAN. In our study, we are investigating the learning capability and computational efficiency of machine learning models on a large and diverse epigenomics dataset. For this purpose, we include all distal regulatory elements (e.g., silencers, promoters, and insulators) as positive samples. This is a more challenging problem because of the diversity of the different types of regulatory elements. We are also the first to identify that CNN is the best-of-breed classifier for this problem domain, due to its ability to capture the spatial abstractions from the input. Further, none of the prior approaches quantify the speedup of the various classifiers, relatively on CPU and GPU; ease of speed up by accelerators being an attractive attribute of neural networks. Finally, fast training, while less critical in some domains, is important in genomics because of the need to quickly retrain algorithms, with newer datasets, a product of rapid technology advances.

### ML Background

The SVM paradigm, originally designed for such binary classification problems, has an impressive geometrical interpretation of discriminating one class from another in a multidimensional input space using a maximum-margin hyperplane [34]. It is commonly known that the SVM paradigm is amenable with the regularization framework, where we have a data fit component ensuring the model’s fidelity to the data, on the one hand, and a penalty component enforcing the model’s simplicity on the other [35*,*^36^]. The SVM methodology is based on a solid theoretical foundation, with the core of the algorithm being a quadratic programming problem, separating support vectors (“supporting” the decision boundary) from the rest of the training examples. Specifically, a linear SVM finds a linear decision boundary with the maximum-margin separation between the support vectors of the two classes in the dataset. Further, for overcoming model bias in a complex data landscape, SVMs can be versatile, deploying different kernel functions. While commonly used kernels are polynomial, radial basis function (RBF), and sigmoid, custom kernels can also be defined using a Gram matrix (Gramian matrix or Gramian), which is a Hermitian matrix of inner products. In essence, a kernel SVM projects data from the input space into an higher-dimensional feature space (can be infinite-dimensional). The exact form of the function is determined by the type of kernel used, we use the RBF kernel. Kernel SVM then finds the hyperplane in that space to “linearly” separate the positive and negative training examples. Theoretically, it is always possible to find such a perfect hyperplane in the infinite-dimensional space. However, empirically, finding the best hyper-parameters is time consuming and even a single run of the kernel SVM algorithm can range in complexity from *O*(*n*^2^) to *O*(*n*^3^) [37], which makes it prohibitive for training a large dataset. Thus, while SVMs are powerful tools, their compute and storage requirements increase rapidly with the increase in the number of training vectors.

Another approach to build a robust classifier is through ensemble methods. Instead of learning a single strong classifier, ensemble methods try to construct multiple weak learners to solve the same problem, random forest being a prominent member of this family. Random forest been proven to be more resilient to overfitting because of its technique of compiling multiple weak learners. However, they are not suitable for large-scale datasets since the memory requirement grows as the function of training set size [41]. Recently, deep learning [38] has emerged as a powerful tool in the machine learning community, abetted by the volumes and diverse types of datasets. Several theoretical studies [39*–*44] have shown that deep learning approaches are able to learn high-level abstractions from data using architectures consisting of multiple layers of non-linear processing units using a variety of activation functions, with the increasing depth of the layers increasing the power of abstraction, but only up to a certain point. Although the success of DNNs is attributed to the hierarchy introduced by the hidden layers making it sort of a hierarchical processing pipeline, there is a tradeoff between the accuracy achieved by this process and the time taken to train this network. Common examples of the areas in which deep learning has been successful include image classification [21], automatic speech recognition [47], and natural language processing [48]. More recently, deep learning has gained traction in several prediction problems in bioinformatics, such as structural predictions of proteins [49] and of RNA-binding protein targets [50], of RNA splicing predictions [51], and of genome annotations with some degree of interpretability [54]. We select two variants of DNNs, the vanilla Deep Neural Network (DNN) architecture and Convolutional Neural Networks (CNN), to solve the distal regulatory element prediction problem.

## Results

We preface this section with our evaluation metrics and data-preprocessing techniques.

### Performance Metrics and Data Preprocessing

In this subsection, we start with the benchmarking metrics used for the tools we developed in AIKYATAN. We then move on to the data preprocessing techniques that were used to refine the raw data to be used as inputs to our classifiers. *Performance Metrics*: We use Validation Rate (VR) to benchmark AIKYATAN. The standard benchmarking metrics, e.g. precision-recall (PR) metrics are not valid here since there are regulatory sites that have not been experimentally mapped, beyond p300, NANOG, SOX2, OCT4 binding sites or TSS. In order to use the more conventional benchmarking metrics, we would have to evaluate performance on all these sites that are unaccounted for, which are not experimentally mapped and hence unknown. This is in line with the observation made by other enhancer prediction methods such as RFECS and EP-DNN [11]. Thus, we needed to modify the performance metrics used in AIKYATAN.^2^ RFECS the validation rate (VR) metric by validating a classification against criteria mentioned below. If a location has histone modification enrichment signatures similar to that of an enhancer (or a distal regulatory site in our case) and a prediction is made at that location, we can say that the classification is valid, given that it is located sufficiently close to a known enhancer marker or to an open chromatin site, which means that the site can be exposed to regulatory factors. Following are the criteria designed for VR-based evaluation of the algorithms.

- If a classified regulatory site lies within 2.5 kb of a true positive marker (TPM), then the classification is “validated”, which we also refer to as “gray area”. This is because this site is either a known (experimentally validated) regulatory site, or an unknown regulatory site that overlaps with open chromatin and we can assume to be a regulatory site.
- Otherwise, it is deemed “invalid”, which means it is either a TSS or an Unknown and we know for a fact that the prediction is incorrect.

**Fig. 1:**
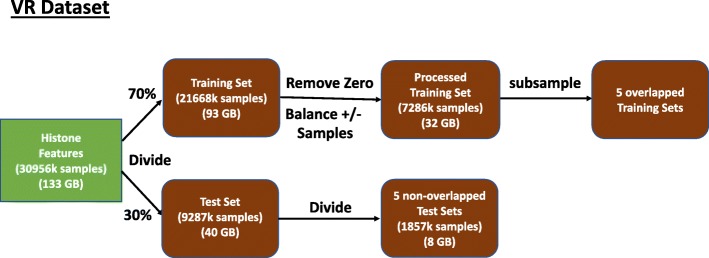
The pipeline for generating Training and Test Sets for VR dataset

*Data Preprocessing*: Figure 1 represents the process of generating the VR dataset. We found that a large fraction of samples (8 percent) have no histone modification signal, which can be seen as all feature values being zero. Such samples provide no meaningful information for training the models, so we remove them from our training set. In addition, the number of positive and negative samples in the dataset is not balanced, with positives samples being in the minority (22% of the total dataset). To avoid modeling bias in favor of the majority class, we balance datasets by subsampling from the much larger number of negative samples. Figure 1 describes the pipeline for generating training and test sets from the raw histone modifications’ input. To reduce the variance, we divide the test set into five non-overlapping test sets and also generate five overlapping training sets for each training set size. The five training sets are paired with the five test sets with a one-on-one correspondence. The final VR numbers are averaged from five train-test pairs for each experiment.

### Empirical Results

We designed experiments to evaluate the training time and prediction accuracy for the different classifiers in AIKYATAN. The machines’ specifications are listed in Table 1. We used Keras [26] as the frontend, with Theano [27] at the backend, to develop our deep learning models. Thus, our infrastructure runs on a Python backend, which is advantageous for ML algorithms as it can benefit from the rapid progress in Python libraries, compared to the development in Matlab or C/C++.

**Table 1:** Computational specifications of machines used for the experiments

Environment	CPU	Number of Cores	GPU
GPU machine	Intel(R) Core(TM) i7-2600 CPU @ 3.40 GHz	4	NVIDIA Telsa K40
CPU machine	Intel(R) Xeon(R) X3430 CPU @ 2.40 GHz	4	N/A
GPU machine	Intel(R) Xeon(R) Platinum 8168 CPU @ 2.70GHz	24	NVIDIA P100

#### **Deep learning models demonstrate faster computation time even on CPU**

**Fig. 2:**
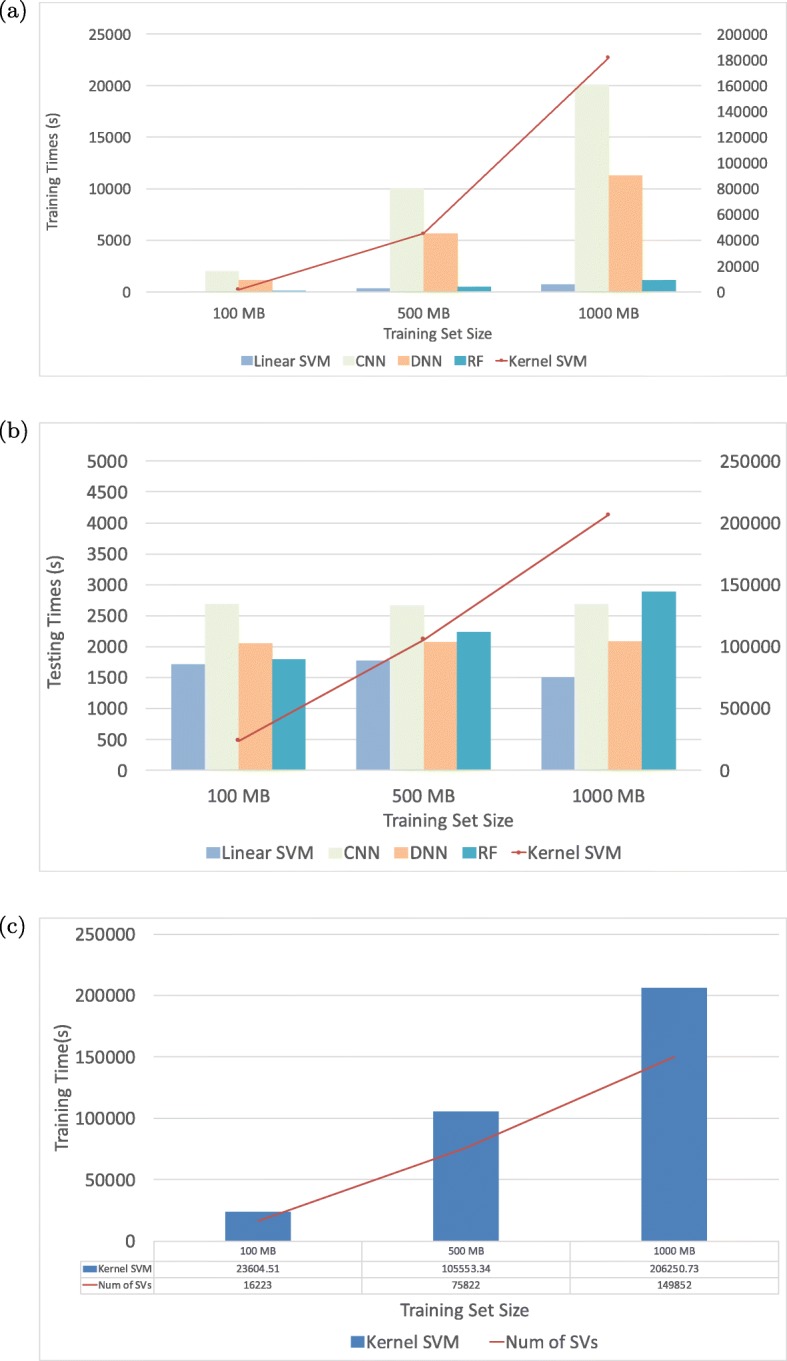
Comparison runtime for Aikyatan. Figures 2a and 2b show the training and testing times using CPU for the models, with varying training set sizes. As shown in Figure 2**a**, linear SVMs, DNNs, and CNNs training times scale approximately O(n) while random forests traing time grows at the rate of O(nlog(n)) and kernel SVMs training time grows at the rate of O(n2.2), where n denotes the number of training samples. As in Figure 2**b**, linear SVMs, DNNs, and CNNs testing times remained constant, whereas random forests testing time grows with the rate.(mn), where m denotes the number of trees, and kernel SVMs testing time grows rapidly as training size increases, with corresponding increase in SVs. Figure 2**c** shows the relationship between the number of SVs obtained from the training set and the testing time for the kernel SVM. For the kernel SVM, the testing time grows linearly with SVs

Without a doubt, it is important that a prediction model should give us superior prediction accuracy. However, we also have to take the computation time into serious consideration when choosing a prediction model. Both training and testing times are important metrics for any ML algorithm though traditionally testing time has been considered the more important of the two. However, in the genomics domain, where volumes of new datasets are becoming available, the model will have to be retrained to update itself on a regular basis and therefore we are also interested in the training times. We measure the training time and testing time as a function of training set size for AIKYATAN on the CPU machine. Figure 2a shows the average training times of the five classifiers with various training set sizes. Random forest exhibits *O*(*nlog*(*n*)), where *n* denotes the number of training samples, training time complexity. Linear SVM, CNN, and DNN, have training time algorithmic complexity of approximately *O*(*n*), while for kernel SVM with RBF kernel, it is between *O*(*n*^2^) and *O*(*n*^3^) [37]. For our specific parameter for the misclassification penalty, this is found to be *O*(*n*^2.2^). We find empirically that the training time follows the relation linear SVM < random forest < DNN < CNN ≪ kernel SVM. With the largest training set size in this experiment, 1,000 MB (226k samples), kernel SVM’s training phase takes around 50.5 hours, which is 255.6x, 161.8x, 9.0x, and 16.1x slower than the linear SVM, random forest, CNN, and DNN, respectively. Figure 2b shows the average testing times of the 5 classifiers with various training set sizes. For most ML models, training set size does not affect time required for testing. This is evident from the results for the linear SVM, DNN, and CNN models. However, the testing times for the kernel SVM and random forest *do increase* with training set size Figure 2c. For random forest, the prediction time depends on the depth of trees. In an average case, it is of order *Θ*(*mn*), where *m* is the number of trees. From Fig 2b, we notice that as the training set size grows to 1000 MB, the prediction time is larger than DNN, CNN, and linear SVM. For kernel SVM, the prediction time grows linearly with the number of SVs, as we show in Fig 2b. With the training set size of 1000 MB (226k samples), kernel SVM’s testing phase takes around 57.3 hours, which is 136.9x, 71.4x, 76.7x, and 98.9x slower than a linear SVM, random forest, CNN, and DNN, respectively. Thus, although a kernel SVM has superior prediction performance, the prediction times make it impractical to use, as datasets tend to be very large in our problem domain. To summarize, we have shown that when we use CPU for computation, the training *and* testing times of a kernel SVM are much higher than for the other models and the rate of growth in running time is also higher for a kernel SVM. In the case for random forest, although the time requited to construct model is relatively low, the prediction time is higher than other DNN, CNN, and linear SVMs when the size of training set is large.

*Computation Cost Comparison for CNN, RFECS, and CSIANN* Here, we compare the training and testing time for CNN with RFECS and CISANN on 16 GB training set (3643k samples). We could not deploy RFECS and CSIANN on the CPU machine (X3430 processor) that we used for the experiments with AIKYATAN (specs in Table 1) because of smaller numbers of cores and lower clock rates of the processor. Instead, we ran RFECS and CSIANN methods on the higher-end Platinum 8168 processor with 24 cores. While utilizing all cores on the higher-end CPU, RFECS still takes 45.6 hours for training and 1.78 hours for testing while AIKYATAN-CNN takes 9.13 hours for training and 0.27 hours for testing. Thus, RFECS’ training time is about 5X that of ours^3^. For CSIANN, a bottleneck of the model lies in the high computation cost of the inversion of the large matrix, *O*(*d*^3^) where *d* is the dimension of features and usually *d*>>1, during the Fisher Discriminant Analysis. We failed to finish the training of CSIANN within a week using CPU. Thus, we put the matrix inversion computation task into a P100 GPU while other computations remain on CPU for CSIANN. After this modification, CSIANN still takes 31 hours for training and 1.5 hours for testing, 3X times slower than our CNN. In summary, CNN modeling takes less time to train than both RFECS and CSIANN and is also easily amenable to speedup by GPUs. For the next experiment, we investigate how much we can speed up both training and testing through the use of a GPU.

#### **Deep Learning Models can leverage GPU-based accelerators**

**Fig. 3:**
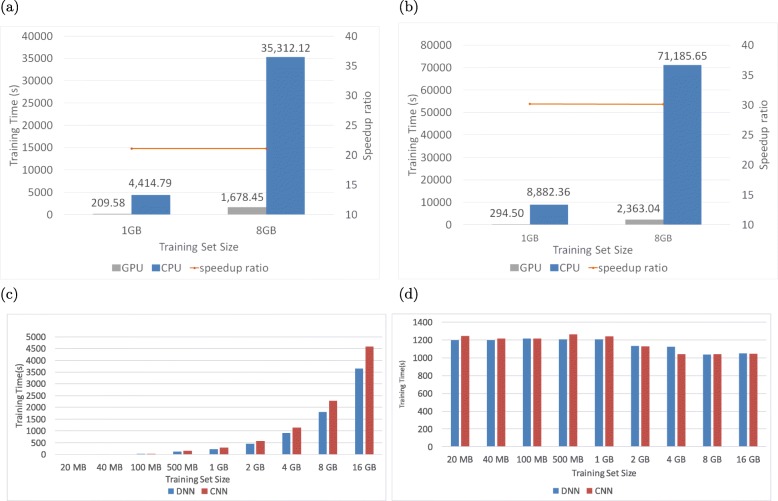
Training and Testing times and GPU speedup of DNN and CNN models. Figures 3**a** and 3**b** show the speed-up ratio for DNN and CNN, respectively. The orange line represents the speed-up ratio, training time using CPU divided by training time using GPU, for training set sizes varying from 1 GB to 8 GB. The speed-up ratio remained constant and the speed up is around 21x for DNN and 30x for CNN, respectively. Figures 3**c** and 3**d** shows how training time and testing time grows as training set size increases for DNN and CNN, when deployed on GPU. We fixed DNN and CNN architectures among all training sets and the number of learning epochs to be 50. Both DNN and CNN training times grow linearly when deployed on GPU

The computation in a neural network can be decomposed into multiple matrix operations, which have the Single Instruction Multiple Data (SIMD) characteristic. These operations are therefore well suited for exploiting the parallelism that is available on GPUs. In this experiment, we quantify how much speedup is possible for AIKYATAN DNN and CNN variants by executing them on a GPU. We fixed the model architectures and used the same number of training epochs, which is 50, for both DNN and CNN and trained on different training set sizes. In order to train on a larger dataset, we used the datasets used for VR metrics in this experiment. We first examine the speedup ratio of using GPU over CPU. Figure 3a and b show the training times for DNN and CNN respectively. For DNN, using GPU is 21x faster than using CPU, while for CNN, it is 30x faster. This can be explained by the fact that CNN training involves a greater number of matrix operations for the convolution phase and thus the CNN operations can more effectively leverage all the GPU cores.

Next, we examine the training time and testing time for DNN and CNN on GPUs for different training set sizes.

Figure 3c and Fig 3d shows the training and testing time on GPU for DNN and CNN using varying training set sizes from 500 MB (133k samples) to 16 GB (3643k samples). The training and testing time on GPU behaves similar to the training and testing time on CPU for both DNN and CNN in that the training time grows linearly with the training set size and the testing time remains constant no matter how the size of training set size grows. With the largest training set size of 16 GB, DNN takes around an hour and CNN takes 1.27 hours for training on GPU. Regardless of training set sizes, CNN’s training time relative to DNN’s remains constant, at approximately 1.2. CNN’s testing time relative to DNN’s also remains constant and the two are approximately equal.

#### **CNN achieves superior performance in prediction capability and time compared to state-of-art methods**

First, we show the prediction performance of our CNN with state-of-art methods, e.g., RFECS [31] and CSIANN [30]. Because of the high dimensionality of the training data, both RFECS and CSIANN managed to make the computation tractable by using only a subset of histone modifications for learning. Furthermore, CISANN reduces the dimensionality of features using Fisher’s Discriminant Analysis (FDA). In contrast, we aim at demonstrating our computational model is not only able to consume high-dimensional data but also able to learn intricate non-linear features from them resulting in higher expressiveness. Toward achieving a fair comparison, we used our dataset (24 histone modifications instead of a subset) and applied it to RFECS and CSIANN. Again, we selected RFECS and CSIANN as two representative leading edge sophisticated models that use similar epigenomics datasets as AIKYATAN (as inputs to the model) and known to be sophisticated classifiers while being distinct. Table 2 shows the average VR and the standard deviation of VR on a 16 GB training set for CNN, RFECS, and CSIANN. CNN achieved 1% higher VR than RFECS even though it has already achieved a reasonable VR of 96.65%. CSIANN made two simplifications. First, dimensionality-reduction techniques were used so that coarser features were used for the learning process. Second, only one hidden layer was used for its neural network model. With these two simplifications, CSIANN, performed better than random guessing, but was not able to generalize well on our distal regulatory elements’ prediction problem. Finally, CNN is the most insensitive to the changes in dataset, which is shown in Table 2. The standard deviation of VR derived from the five 16 GB datasets is the smallest, compared to RFECS and CSIANN.

**Table 2:** VR Numbers on 16 GB (3643k samples) training set for CNN, RFECS, and CSIANN

	CNN	RFECS	CSIANN	Random
Avg. VR	0.9786	0.9665	0.5715	0.2265
Std. VR	0.0068	0.0100	0.1754	NA

**Fig. 4:**
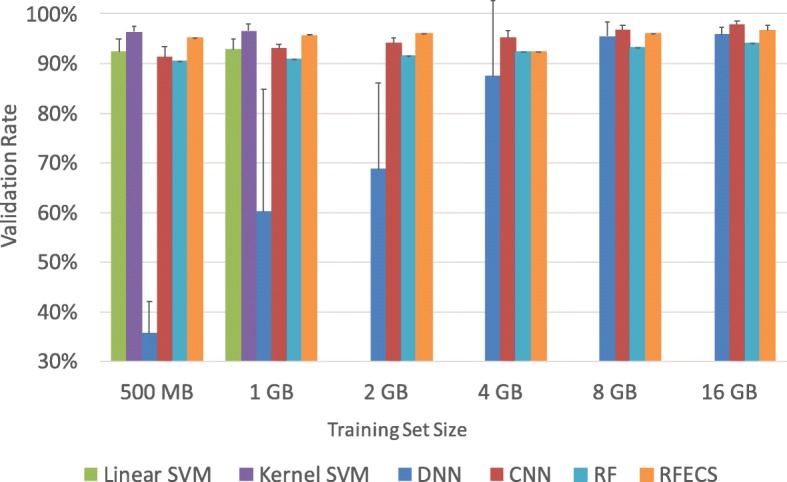
Average VR performance are shown for Aikyatan. To obtain a larger data set size, unlike in RFECS, where the training set only contains peaks, we include gray area into our training set. By varying the threshold that is used to turn the raw real-valued prediction into one of the two classes, we can generate a VR curve where X-axis is the number of samples predicted as positive and Y-axis is the portion of these predicted positive samples that are validated, i.e., the validation rate (VR). In order to compare the prediction performance across the ML models, we control for the same number of predictions across these models. In order to find the specific number of predictions, we obtained the target number of predictions from RFECS where the best validation in its original data set is for around 100K predictions. Since we took 70% of the original data set as the training set and 30% as the test set and further divided test sets into 5 non-overlapping test sets, our target number of predictions becomes 6000 in each sub-sampled test set

Next, we are also interested in how the performance grows as a function of training set size. We investigate our AIKYATAN’s prediction performance with RFECS [31]. We do not do further analysis for CSIANN because not only other methods significantly outperform its inference capability but also its high computation cost due. Figure 4 shows the average VR, benchmarking the predictions of AIKYATAN vis-à-vis competition. *Algorithmic Performance*: Both kernel and linear SVM achieve a high VR for small training set sizes, outperforming deep learning variants. However, as the training set size becomes larger, the rate of improvement for both linear and kernel SVM is smaller than for deep learning approaches, especially DNN. Further, the variation of DNN performance on smaller datasets is high, indicating that the DNN model is not stable at this size. This phenomenon occurs because of the large number of learning parameters of a DNN. But as the training set grows, the DNN’s performance becomes stable and outperforms linear SVM. Looking at the trend, one would expect that a kernel SVM can achieve higher VR with larger training set sizes. However, due to a kernel SVM’s high computational cost, we could not train the model for a dataset size larger than 1 GB (230k samples) in an acceptable time.

On the contrary, the lower computational cost of DNN and CNN allows us to train them using increasingly larger training sets as more and more data becomes available for building the model. We find that the VR performance of deep learning approaches continues to improve with increasing training set sizes. Using 16 GB (3643k samples) training sets, DNN can achieve similar VR to a kernel SVM, while CNN can outperform a kernel SVM, requiring less time for both training and testing phases, which we have already quantified in previous experiments. We also test the performance for random forest. As we can see, although random forest is more stable than other methods, it does not increase much prediction performance as training set size grows. When trained on the largest data set, random forest only achieve 94 % validation rate, 1.7 and 3.8 worse than DNN and CNN respectively. RFECS improves the performance for random forest, at the smallest dataset in this experiments it starts with 95% and reach to 96.65%. However, the VR is still at the same level with kernel SVM and 1.2% worse than CNN. Ranking the Algorithms in AIKYATAN: To rank average VR performance among the four ML models in AIKYATAN, we perform statistical significance tests to compare (1) linear SVM and kernel SVM on 1 GB training sets, (2) kernel SVM on 1 GB training sets versus DNN on 16 GB training sets, (3) kernel SVM on 1 GB training sets versus RFECS on 16 GB training sets, and (3) DNN and CNN on 16 GB training sets. (4) DNN and random forest on 16 GB training sets. For (1), (3), and (4) we use paired one-tailed t-testing since they are trained using the same group of training sets, whereas for (2) and (3), we use unpaired one-tailed t-testing since they use different groups of training sets. We found that all of the p-values are smaller than 0.05, with the exception of case (2) and (3). We conclude that CNN outperforms the other five models; that kernel SVM, RFECS, and DNN are at the same level; DNN outperforms random forest; and that the linear SVM’s performance is the worst because of the bias (underfitting).

## Discussion

Kernel SVM has emerged as a popular general-purpose ML model and has been used successfully in many domains, especially because of its solid theoretical foundations, based on Vapnik–Chervonenkis theory (VC theory [34]). The first results in the field of discrimination, exposed in Vapnik and Chervonenkis (1971), dealt with the computation of dichotomies with binary-valued functions. However, Kernel SVM’s major drawback is its high time complexity to train the model, which is a quadratic to cubic function of the number of training samples. This puts a strain on how much data can be used to train the model, which can lead to situations where the learned model is not discriminating enough to capture all the nuances in the data. In the genomics area, increasing amounts of data are becoming available, and therefore, there is the possibility of using larger and larger amounts of training data to improve a classifier’s accuracy. This led us to consider deep learning models for the problem of predicting distal genomic regulatory sites. However, since long training times are a bottleneck for deep learning algorithms, we use GPU accelerators for faster execution of our neural network models. From other domains, such as computer vision applications of image recognition and classification, it is known that CNN converges faster than DNN if the shift invariant property of the pattern holds. We hypothesized that stemming from the three-dimensional folding abilities of a genome and the image-like properties of the histone modification signals, the translational invariance property also holds for our problem. So, we evaluated CNN architectures alongside DNN and verified this fact. Our results hold promise for the use of deep learning approaches for high-dimensional and diverse genomic datasets. While we have used single-node executions here (for both CPU and GPU implementations), it is possible to use distributed deep learning frameworks, such as TensorFlow [63] and PyTorch [64] as the data volumes and heterogeneity become more substantial. Given that AIKYATAN is the first algorithm of its kind classifying DREs, many of which are yet to be studied in detail, we believe our algorithm can reduce the noise and discover patterns in new types of DREs plus capture the nuances in existing classes of DREs, for example, in enhancers and their sub-types.

## Conclusions

In this study, we demonstrate how we formulate histone modification signals as snapshots and demonstrate how local feature extraction and the shift-invariant property of a CNN can apply to histone modification signals and combinatorial epigenomic features. Empirical results demonstrate that CNN has superior generalization performance, achieving a validation rate of 97.9% or higher, compared to standard DNN, linear SVM, kernel SVM as well as the state-of-the-art methods, such as CSIANN and RFECS. Moreover, we give empirical results on training and testing times. With GPU enabled, CNN’s training time is sped up by 30x over a CPU. With the largest training set size in training time comparison of AIKYATAN, 1,000 MB (226k samples), kernel SVM’s training phase takes around 50.5 hours, which is 255.6x, 161.8x, 9.0x, and 16.1x slower than the linear SVM, random forest, CNN, and DNN, respectively. Overall, taking into account the expressiveness of the ML models and the computational efficiency, we conclude that Distal Regulatory Element prediction task favors CNN due to its high expressiveness and ease of accelerating its computation.

## Methods

### A. Overview

**Fig. 5:**
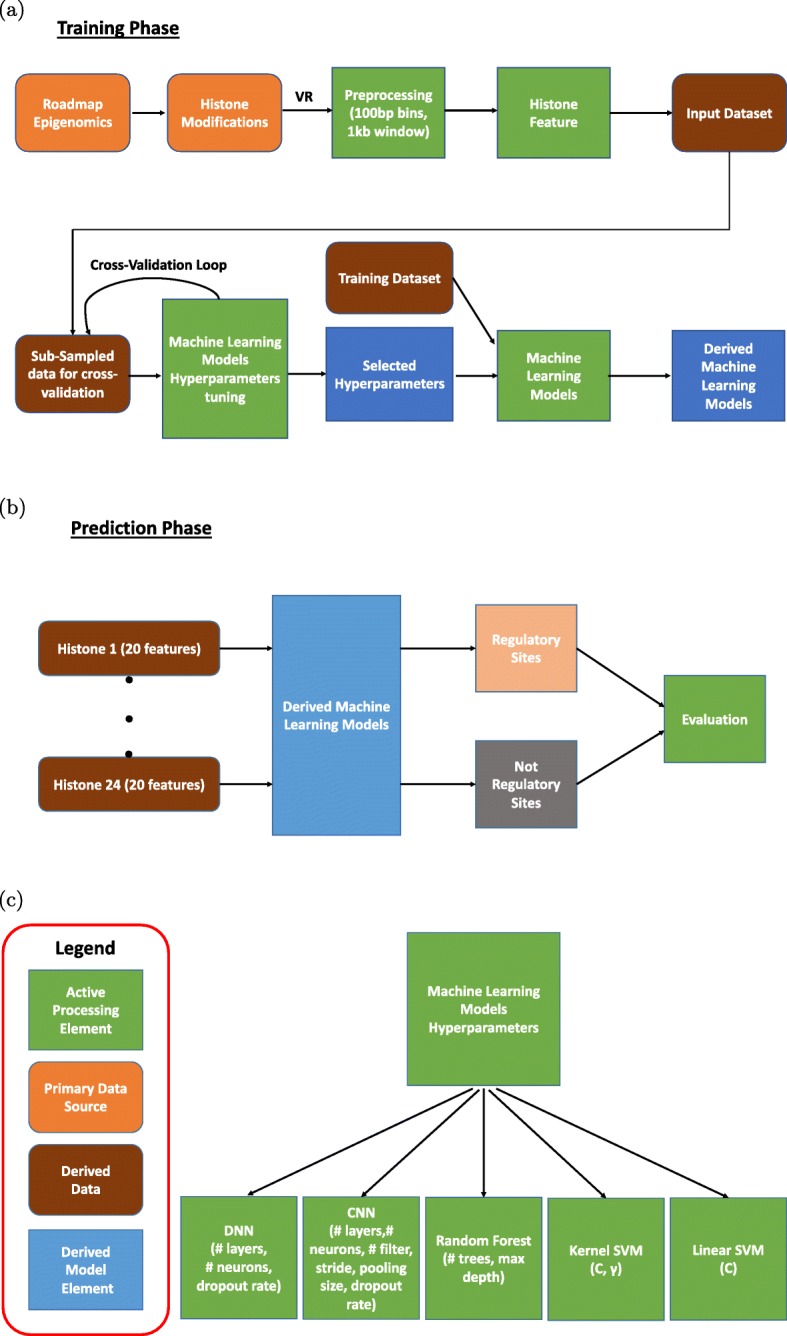
An overview plot describing five machine learning (ML) models training and testing phases. Figure 5**a** describes the training phase for four ML models. Figure 5**b** describes the prediction phase. After having tuned the hyperparameters for each model, we evaluate its performance using the validation-rate (VR) metric. Figure 5**c** describes the legend we use and the hyperparameters tuned for each model

Figure 5a, b, and c represent an overview of AIKYATAN’s training and testing phases. Our AIKYATAN suite includes a linear SVM model, a radial basis function (RBF) kernel SVM model, random forest, and deep learning variants, DNN and CNN for the task of predicting DREs in the human embryonic cell line (H1), a tier 1 ENCODE project cell type. To obtain the feature vector for each genome position, we use histone modification signatures as input features. Our binary classification task then is as follows: given histone modification signatures at genome location *i*, predict whether genome position *i* is a distal regulatory site or not, *i.e.*, distal to promoters or TSSs.

### B. Epigenomic datasets

Histone modification signatures: We use 24 histone modifications for our prediction task. The data was obtained from the NCBI database under NCBI GEO accession number GSE16256. The 24 histone modifications are as follows: H2AK5ac, H2BK120ac, H2BK12ac, H2BK15ac, H2BK20ac, H2BK5ac, H3K14ac, H3K18ac, H3K23ac, H3K27ac, H3K27me3, H3K36me3, H3K4ac, H3K4me1, H3K4me2, H3K4me3, H3K56ac, H3K79me1, H3K79me2, H3K9ac, H3K9me3, H4K20me1, H4K5ac, and H4K91ac, in H1, which were generated as a part of the NIH Epigenome Roadmap Project [52]. These histone modifications comprise of a superset of all that are hypothesized to be relevant biologically to the presence (or absence) of regulatory sites [31]. The ChIP-seq reads of these histone modifications give us their enhancement level. These were binned into 100 base pair (bp) intervals and normalized against their corresponding inputs by using an RPKM (reads per kilobase per million) measure [53]. Multiple replicates of histone modifications were used to minimize batch-related differences and the replicates’ RPKM-levels were averaged to produce a single RPKM measurement per histone modification. This averaged RPKM enrichment level of a histone modification is its signature. For any given location, the histone modification signatures within 1000 bp of that location are used as input to the models. A window of 1000 bp incorporates ten 100 bp bins on each side. With 20 bins for each of the 24 histone modifications, the input comprises 480 features in total. Included locations: For training and testing, the positive set includes all the p300 binding sites, cell type-specific Transcription Factor Binding Sites (TFBS) (NANOG, OCT4, and SOX2), and DNase I Hypersensitivity Sites (DHS), which are at least 1000 bp away from the nearest known Transcription Start Site (TSS). Since p300 co-activators, DNase I, and Transcription Factors (TFs) also bind to TSS, which are not distal regulatory elements, we only considered the binding sites that are distal to known TSS sites as positives. The remaining locations were considered as negatives. Narrow DHS peaks were downloaded from UCSC’s ENCODE site. [54] The accession numbers: GSE37858, GSE18292, and GSE17917, contain genome-wide binding data for H1 p300, NANOG, OCT4, and SOX2. p300 and TF peaks were determined using the MACS peak-calling software, with default p-value cutoffs. ChIP-seq input files were used as treatment or background.

### C. Machine learning models

In this work, we selected a suite of ML protocols under the banner AIKYATAN^4^, e.g., linear SVM, kernel SVM, random forest, DNN, and CNN, specifically targeted to this problem and using chromatin-based features, namely, 24 histone modifications’ signatures as feature set, for the classification task. The description of SVMs, random forest and the corresponding hyperparameter tuning procedure can be found in the Supplementarty materials. A high-level goal of our work is to optimize individual “algorithmic motifs” or “kernels” recurring in computational genomics algorithms and then stitch together an optimized library of kernels for specific genomics applications, as envisioned in the domain-specific library (DSL)—Sarvavid [59]

#### Deep neural network model

**Fig. 6:**
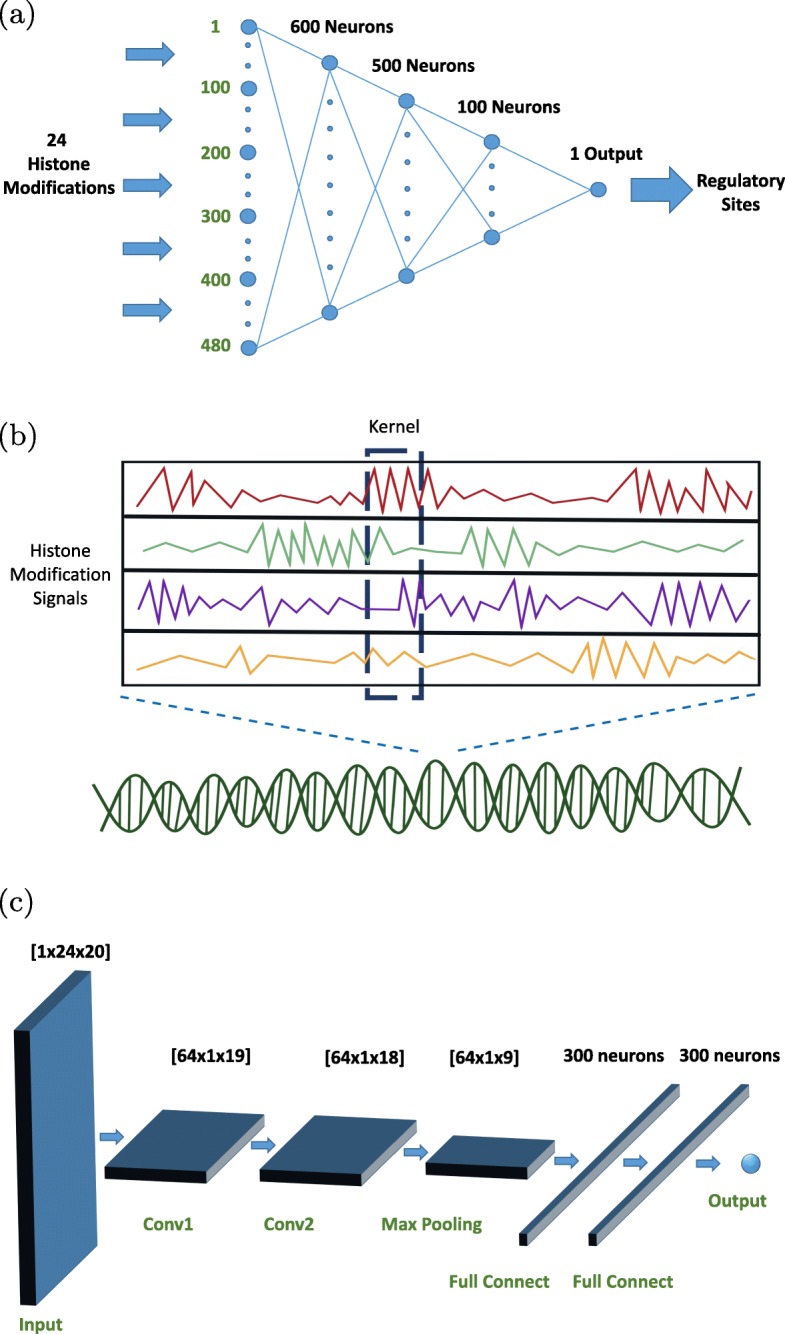
Figure 6**a** shows the DNN architecture. It takes 24 histone modifications (each has 20 features) as input and predicts whether a genomic location is a distal regulatory site or not. There are three hidden layers and one output layer. Between each hidden layer, we used PReLU as activation function and dropout with rate 0.3 between each hidden layer, to prevent overfitting. Figure 6**b** gives an illustrative example of row-wise stacking of histone modifications used as inputs to our CNN model. As shown in Figure 6**b**, each location has various histone modification signals, represented by zigzag lines with di.erent colors in the figure. For illustration purposes, we only represent four histone modification signals. By stacking these signals row-wise, these signals are captured as snapshots of informative features of the genome at each location. Similar to standard RGB images where channels provide di.erent color features, each type of histone modification signal provides unique information to the model. Since the patterns of those signals are quite di.erent across di.erent types of histone modifications, removing any subset of them could result in information loss. With the proper design of the convolution kernel, where the height can cover all signals, the convolution kernel can extract local features to the next layer of the designed CNN. The width of the kernel should not be too large. Too wide a kernel would result in the kernel convolving remote features that are irrelevant to characterizing the local information. Figure 6**c** shows the CNN architecture. The input is in 2D form with each row representing one histone modification feature. After each convolutional layer, it has PReLu layer (due to the space constraint, we skipped showing them in the Figure). After Max-Pooling for down sampling, CNN connects two layers of fully connected neurons, each layer has 300 neurons, and finally connects with output. To prevent overfitting, we also add dropout with rate 0.5 between Max-Pooling and first fully connected layer and between first and second fully connected layer, and dropout with rate 0.3 between the second fully connected layer and output layer

The DNN architecture has 480 inputs and and 1 output, applying the *PReLu* (Parametric ReLu [55]) activation function for each neuron, which is essentially a Leaky ReLu but with a learnable coefficient to tackle the dying ReLu problem in the vanilla ReLu function. The tuned-DNN architecture has three hidden layers, with 600 neurons in the first layer, 500 in the second, and 400 in the third. To prevent overfitting, *dropout* was applied between each hidden layer, with a dropout rate of 0.3. We use mean squared error as the loss function. We experimented with the following optimizers: *RMSProp* [56], *Adadelta* [57], *Adagrad* [58], and *Adam* [59]. We found that the *RMSProp* [56] optimizer worked best for this DNN architecture. The DNN architecture is shown in Fig 6a.

#### Convolutional neural network model

CNNs have tremendously improved the prediction performance of image-classification tasks. This improvement comes from the following attributes of CNNs.

- CNNs are able to perform local feature extraction through the design of specific filters that can pick up target features from the images, and at scale, the parameters such as stride length and filter size can modify the rate at which these target features are detected from the images.
- CNNs demonstrate a shift invariant property, which means the exact position of the features does not matter and this comes from the pooling of the features in the pooling step, a useful artefact of which is the dimensionality reduction that occurs in the process.
- CNNs perform non-linear transformation of the input through the use of various activation functions. Since the third characteristic is similar to traditional neural networks, we only describe *local feature extraction* and *the shift-invariant property* in greater detail. **Local feature extraction:** Images have structures, with increasing levels of complexity starting with local features of the image and moving on to more abstract, global features. Distinct from the standard fully-connected neural network that treats each pixel position as an independent variable, the kernel of the convolutional layer in a CNN looks at a small region of the input (receptive field) at a time and extracts meaningful features locally from the input (initially). The subsequent convolutional layers hierarchically extract higher-level features from the previous layers’ output and the process carries on with the ability to extract higher-order abstractions with increasing network depths. Now these kernels are essentially an array of numbers (called weights or parameters of the filter) and these “kernel weights” are adjusted throughout the learning process. At the end, these kernels are capable of extracting relevant features for increasing the prediction performance for the task at hand. **Shift invariance:** There are two invariant properties of CNNs: *location invariance* and *translation invariance*. First, since the weights of a specific kernel are shared when scanning through the local region of inputs, no matter where the object that the model is trying to identify, “scanning” the kernel across the image will produce the same output. In other words, the weight sharing characteristic of the kernel of the convolutional layer allows the learned model to be insensitive to the location of the target object in the image. We call this the *location invariant* property of the CNN. Second, when a kernel scans a specific region of input, it computes the dot product between the learned weights and the local inputs. Thus, if the original input is slightly rotated, the dot product does not change much. The pooling layer essentially performs a downsampling operation to the output of the previous layer. Specifically, it distills the most salient features among the nearby ones to capture snapshots in the images. Thus, no matter where the salient features are located within that region, the pooling operator will pick them up. These two factors contribute to the *translation invariance* property of the CNN.

**Histone modification signals are snapshots of genome:** Typical images have three channels: R, G, and B. Each channel encodes different values for the same location of the image and these values are essential to represent image. One can also only use gray scale to represent images. However, the gray scale images discard the color information. Similar to images, different histone modification signals characterize distinct properties at each genome location. Therefore, by stacking each histone modification feature row-wise with the proper design of filters or kernels, a location-by-location snapshot of the genome is acquired. We give an illustrative example of how we stack histone modification combinatorial signatures for encoding the information into the CNN in Fig 6b. We hypothesize that *the information extracted from histone modification snapshots can be well characterized by the CNN model* due to the following reasons. First, the histone signals may be slightly transformed due to the sampling techniques. Those nuances should not affect the output of the learned model. Second, the location of histone modifications signals in the snapshot should not affect the prediction outcome. And third, the permutation of histone modification signals should not change the prediction outcome. We believe that CNN could generalize well from histone modification snapshots since it can perform local feature extraction and can preserve the shift invariant property. Our empirical results support our hypothesis.

**Sensitivity analysis on the hyperparameters’ tuning space:** A valid concern when using deep learning models is that the search space for hyperparameter tuning is too large to generate a specific architecture for a specific problem statement. However, through our analysis for tuning the hyperparameters, we find that the searching is tractable and can be explained by standard learning theory [34]. Specifically, we test the size of the kernels of the convolutional layers and the window size of the pooling layer. We find that the higher the number of kernels, the better the validation rate is, up until 128 kernels. This is because the designed CNN requires enough number of kernels to extract distinct features, in order to construct more nuanced outputs for the next layer. However, if the number of kernels exceeds 128, those additional kernels become redundant, resulting in the CNN overfitting to the noise in the features, as is typical in the genomics domain. We leave the details of the sensitivity analysis on these hyperparameters in supplementary Figure S2a, S2b, and S2c.

**Final CNN architecture:** Our final CNN architecture after performing sensitivity analysis is shown in Fig 6**c**. The 480 input features are reshaped into two dimensions, with 24 rows of histone modifications and 20 columns of features for each histone modification. The first convolutional layer uses 64 kernels, with 24 rows and 2 columns, with stride size of 1 to scan through the input, forming the output volume of the first convolutional layer as [64 ×1×19]. The second convolutional layer uses 64 kernels, with 1 rows and 2 column, with a stride size 1, forming the volume [64 ×1×18]. Each convolutional layer connects with *PReLu* layer for thresholding the output from convolutional layer, retaining the same output volume as its previous convolutional layer. The *Max-Pooling* [60] uses pool size [1 ×2] for downsampling. After downsampling, it connects with two fully-connected layers, each with 300 neurons. Finally, the second fully-connected layer connects the last layer with one neuron representing the output layer. We use mean-squared error as the loss function. We tried *RMSProp* [56], *Adadelta* [57], *Adagrad* [58], and *Adam* [59] optimizers and found *Adagrad* [58] to work the best for our model. In order to prevent overfitting, we added dropout at a rate of 0.5 between *Max-Pooling* and the first fully connected layer and between the first and second fully connected layer, and dropout rate of 0.3 between the second fully connected layer and the output layer.

## Additional file

Additional file 1 This file contains the following: a brief summary of SVM and Random Forest models for distal regulatory site prediction, PR Metric and Data Preprocessing, PR Results, 1 Figure that describes the pipeline for generating the PR dataset, 1 Figure that summarizes the PR results, and 3 Figures that summarize the Sensitivity analysis for tuning our Convolutional Neural Network. (PDF 421 kb)

## Data Availability

All source code will be made publicly available at https://bitbucket.org/cellsandmachines/aikyatan.

## Abbreviations

AUC: Area under curve
CNN: Convolutional neural network
DHS: DNase I hypersensitivity regions
DNN: Deep neural network
DRE: Distal regulatory element
GPU: Graphics processing unit
ML: Machine learning
PR: Precision recall
RBF: Radial basis function
RF: Random forest
RPKM: Reads per kilobase per million
SVM: Support vector machines
TF: Transcription factor
TFBS: Transcription factor binding site
TPM: True positive marker
TSS: Transcription start site
VR: Validation rate

## Footnotes

1 Aikyatan (pronounced “Oi-ko-taan”, origin: Sanskrit) meaning a common harmonious chord. This name is an

2 We also have the evaluation results using PR metric. We put the results in the supplement for brevity.

3 We admit that there exists a parallel version of RFECS. However, we could also speed up the computation of our models using multiple CPUs and even GPUs. In our experiments, we already have shown that the computation time for CNN on single CPU is significantly faster than RFECS. Thus, we do not do further comparisons of multi-CPUs or GPUs for run time comparisons.

4 Aikyatan (pronounced “Oi-ko-taan”, origin: Sanskrit)meaning a common harmonious chord. This name is an
