# Supplementary material for: AIKYATAN: mapping distal regulatory elements using convolutional learning on GPU
Source: BMC Bioinformatics. 2019 Oct 7;20:488. doi: 10.1186/s12859-019-3049-1 (PMC6781298; doi:10.1186/s12859-019-3049-1)
Supplement: Supplementary file 1 — This file contains the following: a brief summary of SVM and Random Forest models for distal regulatory site prediction, PR Metric and Data Preprocessing, PR Results, 1 Figure that describes the pipeline for generating the PR dataset, 1 Figure that summarizes the PR results, and 3 Figures that summarize the Sensitivity analysis for tuning our Convolutional Neural Network. (PDF 421 kb) [file 12859_2019_3049_MOESM1_ESM.pdf]

Supplementary

Machine Learning Models

Linear Support Vector Machine

Given training vectors $\mathbf{x}_i \in \mathbb{R}^d$, $i = 1, \cdots, n$, and a vector $\mathbf{y} \in \mathbb{R}^n$ such that $y_i = \{1, 0\}$, a linear classifier learns the weight vector $\mathbf{w}$. The decision function is $sgn(\mathbf{w}^T\mathbf{x})$. We chose L2-regularized L2-loss SVM to solve our problem, the primal formulation for which is defined as follows:

$$\min_{\mathbf{w}\in\mathbb{R}^d} \frac{1}{2}\mathbf{w}^T\mathbf{w} + C\sum_{i=1}^{n} \max(0, 1 - y_i\mathbf{w}^T\mathbf{x}_i), \tag{1}$$

where C is the penalty parameter for the misclassification error and is thus used to set the regularization level. The objective function of an SVM is optimizing two things: a separating hyperplane with the maximum margin and a reasonable decision boundary for the training set that correctly separates as many instances as deemed necessary, the two of which are contradictory. Therefore, we need to find a balance between the two through our choice of hyperparameters. The latter is what the penalty parameter C does, that is, it affords a penalty for misclassifying an instance. A low C gives a large margin while a high C results in a tighter fit or a smaller margin with lower tolerance for outliers and may potentially overfit on the training dataset.

The dual problem of an SVM, which lends itself naturally to kernels is:

$$\begin{aligned} &\min_{\mathbf{a}} \frac{1}{2}\mathbf{a}^T(Q + D)\mathbf{a} - \mathbf{e}^T\mathbf{a} \qquad (2)\\ &\text{s.t.} \quad 0 \le a_i \le \infty, \quad i = 1, \ldots, n \end{aligned}$$

where, $\mathbf{e}$ is the vector of all ones, D is a diagonal matrix, $Q_{ij} = y_i y_j \mathbf{x}_i^T\mathbf{x}_j$, and $D_{ii} = \frac{1}{2C}, \forall i$.

We used the Linear support vector machine (SVM) module of the scikit-learn Python ML package for the linear SVM model. In order to determine the ideal values for the penalty parameter of the error term, C, we performed a validation experiment and calculated the validation rate on the validation set. This process was executed for each $C \in \{10^{-3}, 10^{-2}, ..., 10^3\}$ and the best model had $C = 10^{-2}$. Then, the model was tested on a significantly larger dataset (the test set), distinct from the training and validation set, and again the validation rate was calculated to determine the model's effectiveness.

RBF Kernel Support Vector Machine Model

Kernel SVM projects the training vector into another feature space $\mathbb{Z}$ by switching Q_{ij} from $y_i y_j \mathbf{x}_i^T\mathbf{x}_j$ to $y_i y_j K(\mathbf{x}_i, \mathbf{x}_j)$, where $K(\mathbf{x}_i, \mathbf{x}_j)$ is a kernel function of choice. In our study, we chose the Radial Basis Function (RBF) kernel, which is defined as follows:

$$K(\mathbf{x}_i, \mathbf{x}_j) = e^{\left(\frac{-||\mathbf{x}_i - \mathbf{x}_j||^2}{2\sigma}\right)} = e^{(-\gamma||\mathbf{x}_i - \mathbf{x}_j||^2)} \tag{3}$$

where $\gamma = \frac{1}{2\sigma}$, and σ is a free parameter. For $\sigma = 1$, the RBF kernel can be expanded as follows using the Taylor series:

$$K(\mathbf{x}_i, \mathbf{x}_j) = \sum_{k=0}^{\infty} \frac{(\mathbf{x}_i^T\mathbf{x}_j)^k}{k!} e^{(-\frac{1}{2}||\mathbf{x}_i||^2)} e^{(-\frac{1}{2}||\mathbf{x}_j||^2)} \tag{4}$$

The feature space $\mathbb{Z}$ that the RBF kernel function is projected to has infinite dimensions. With this mathematical property, it is always possible to find a hyperplane in an infinite-dimensional feature space $\mathbb{Z}$ to separate two classes of data points. We also used the scikit-learn package implementation for our kernel SVM algorithm. We tune the penalty parameter of the error term, C, and the additional parameter γ introduced by the RBF kernel. Now, γ is not strictly an SVM parameter but a parameter introduced by the kernel. γ controls the standard deviation of the Gaussian function that defines the sphere of influence of each training example defined by hyperspheres around them. It can be thought of as the inverse of the radius of influence of the samples selected by the model as support vectors. Smooth models have lower γ values, while complex models have higher γ values. We evaluate the prediction accuracy for every combination of $C \in \{10^{-3}, 10^{-2}, ..., 10^3\}$ and $\gamma \in \{\frac{1}{480} \cdot 10^{-3}, \frac{1}{480} \cdot 10^{-2}, ..., \frac{1}{480} \cdot 10^3\}$. The best model had $C = 10^0$ and $\gamma = \frac{1}{480} \cdot 10^{-1}$.

Random Forest

Since the intuition of how to construct random forest is straightforward, we only provide the reference here for the curious reader [22]. To select the best model, we tried the number of trees from the list $\{10, 50, 100\}$ and the depth of trees from the list $\{50, 100\}$ and selected the best ones for our problem.

PR Metric and Data Preprocessing

Performance Metrics: The PR experiment poses a harder problem because, in reality, there are many more unknown functional sites than just the p300, CBP, NANOG, SOX2, OCT4-binding regulatory sites, or, TSS. We would have to evaluate the performance on all these sites that are, in reality, not all accounted for using ground truth data. These unknown sites are possibly mis-labeled as negative, thus introducing wrong information for the ML models. Distinct from

the PR metric, the VR metric relaxes the "prediction true" condition to account for the nature of the data set. The data set is such that the regulatory sequences generally span a relatively small region of genome and most are smaller than 1000 base pairs (bp), but some sequences can be between 1000 bp and 5000 bp in size. Therefore, the locations in proximity of a regulatory site (identified by a p300, TFBS, or DHS peak) can potentially be positives. We refer to the set of locations within a small window of proximity to all known regulatory sites as the *gray area*. If the prediction classifies a point in the gray area as positive, that is taken to be a correct prediction and contributes positively to the VR. The PR metrics consider peaks instead and since some unknown regulatory sites are being falsely labeled (owing to the lack of ground truth data), PR accuracy becomes lower. In contrast, VR metrics only consider how much of the peaks and gray areas are validated true, and hence, the relaxed condition makes it easier to achieve a higher VR value. Thus, the VR metric has been used in most recent publications [31, 12].

Training and Testing Data sets for PR- and VR-based Prediction Performance: PR and VR target different criteria for prediction performance, with PR predicting peaks, while VR predicting peaks *and* gray area. Thus, we must prepare two different test data sets. Specifically, the test data set for VR should contain peaks and gray area, whereas the test set for PR should only contain peaks. For the training set, VR should contain peaks and gray area whereas PR does not. Next, the data set for PR was generated using a 500 bp sliding window whereas the data set for VR was generated using a 2500 bp sliding window. The reason for PR's smaller sliding window is to narrow down the gray area around the peak for stricter "peak regions".

Data Preprocessing: We also remove the redundant samples (63% of samples have no histone modification signal) and balance the data set (for the PR data set, positive samples comprise only 0.5%) Figure S1a describes the pipeline for generating training and test sets from the raw histone modifications' input. To reduce the variance, we divide the test set into five non-overlapping test sets and also generate five overlapping training sets for each training set size. The five training sets are paired with the five test sets with a one-on-one correspondence. The final PR or VR numbers are averaged from five train-test pairs for each experiment.

PR Results

In this section, we compare precision-recall curve (PRC) for linear SVM, kernel SVM, DNN, CNN, random forest, and RFECS, by measuring average area under precision-recall curve (AUPR) across 5 training sets of the same size. We trained on 50 MB, 300 MB, 600 MB, and 1500 MB datasets, and tested on 7.8 GB test sets for all ML models. Figure S1b shows average AUPR with different training set sizes for Aikyatan. The decision boundary that classifies regulatory sites for linear SVM is a linear hyperplane. If the dataset is not linearly separable, the accuracy suffers. This appears to be the characteristic of our data leading to the worst-in-class performance of linear SVM, especially at larger training set sizes. Another approach to improve prediction performance is using an ensemble of weak learners to solve a single problem. We chose (vanilla) random forest and RFECS as two candidates for this class. In contrast to linear SVM, a kernel SVM can project the features into a higher-dimensional space and can thus model a more complex decision boundary [66]. With regard to a DNN versus linear SVM, a DNN can learn non-linear abstractions, by composing multiple non-linear functions (one for each layer). Therefore, if there is enough data to train the model, and in our case (at 300 MB and higher), a DNN shines. Deep learning approaches generally perform well as the generalization error bound is tighter when the size of training samples is large enough [37], as validated by our results. Compared to SVM models, deep learning approaches get larger improvement in accuracy, with CNN's accuracy increasing faster with increasing training set sizes.

Algorithmic Performance: In our dataset, RFECS outperforms SVMs and DNN and kernel SVM outperform DNN, random forest, and linear SVM for each training set size. DNN's performance is similar to linear SVM at 50 MB and 300 MB but outperforms linear SVM as the training set size increases to 600 MB and beyond. CNN's performance is similar to kernel SVM at 50 MB and 300 MB but outperforms kernel SVM as the training set size increases to 600 MB and beyond. This is likely due to the fact a CNN can capture spatial nuances in the input. To rank average PR performance for these models, we perform statistical significance testing to compare all ML models on 1500 MB training sets. We use paired one-tailed t-testing and get p-values lower than 0.05. Therefore, we conclude that CNN outperforms the other five ML models, shining as best-in-class in our Aikyatan suite; RFECS outperforms kernel SVM; kernel SVM outperforms random forest, DNN; and linear SVM performs the worst, when evaluated using the PR metric. From these results, one might use kernel SVM or RFECS as candidates to solve the classification when not using deep learning approaches. However, as we have discussed previously, the computation cost for kernel SVM is significantly higher, and for random forests, the memory required

to build the classifier is a linear function of the size of training set. Thus, when considering large-scale data analysis, these two methods pose challenges on building satisfiable inference models.

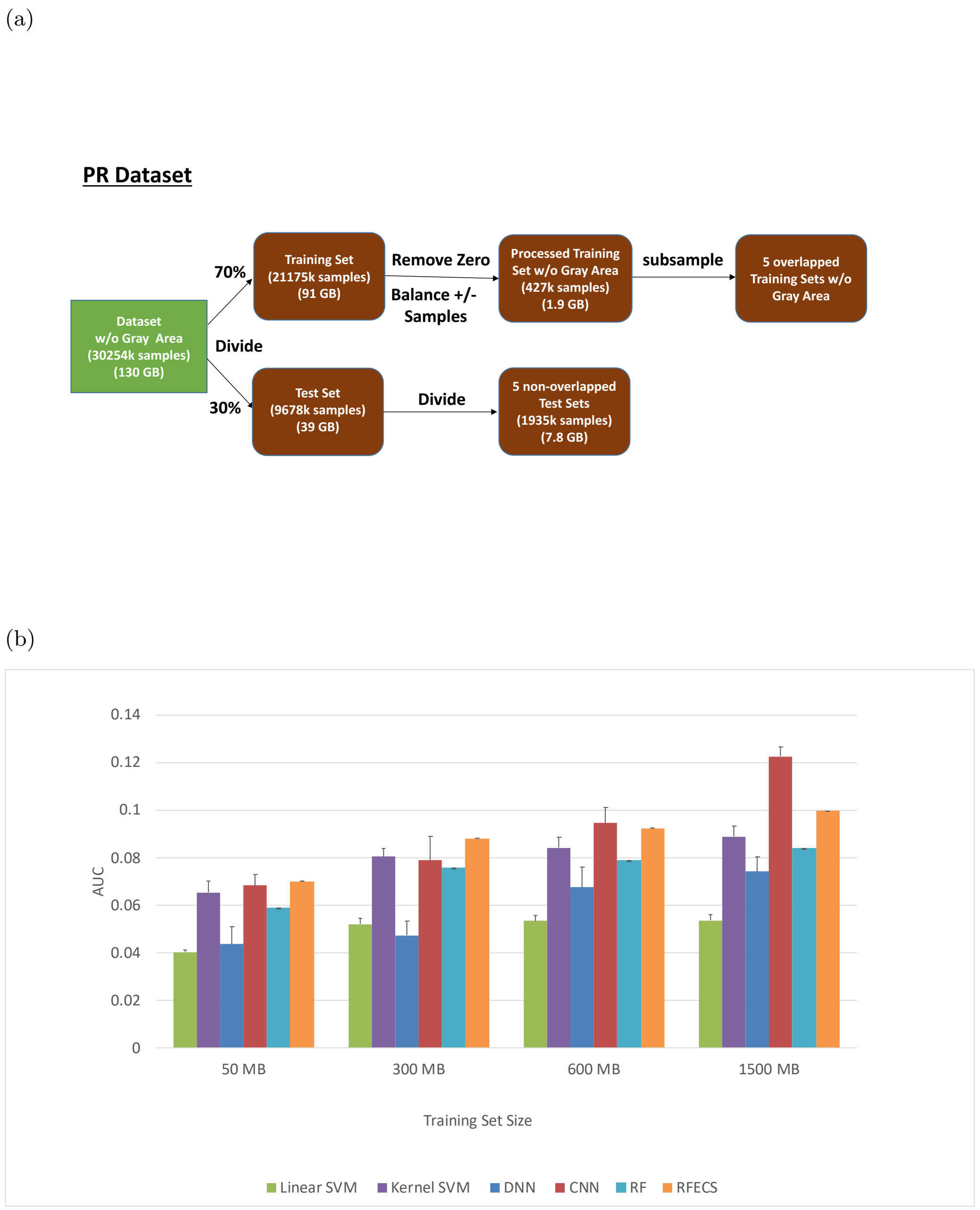

Figure S1: Figure S1a shows the pipeline for generating PR Dataset. Figure S1b presents the PRAUC results for linear SVM, kernel SVM, DNN, CNN, and random forest. Although the overall accuracy downgrades because of the lack of ground truth, we still notice that CNN outperforms other methods.

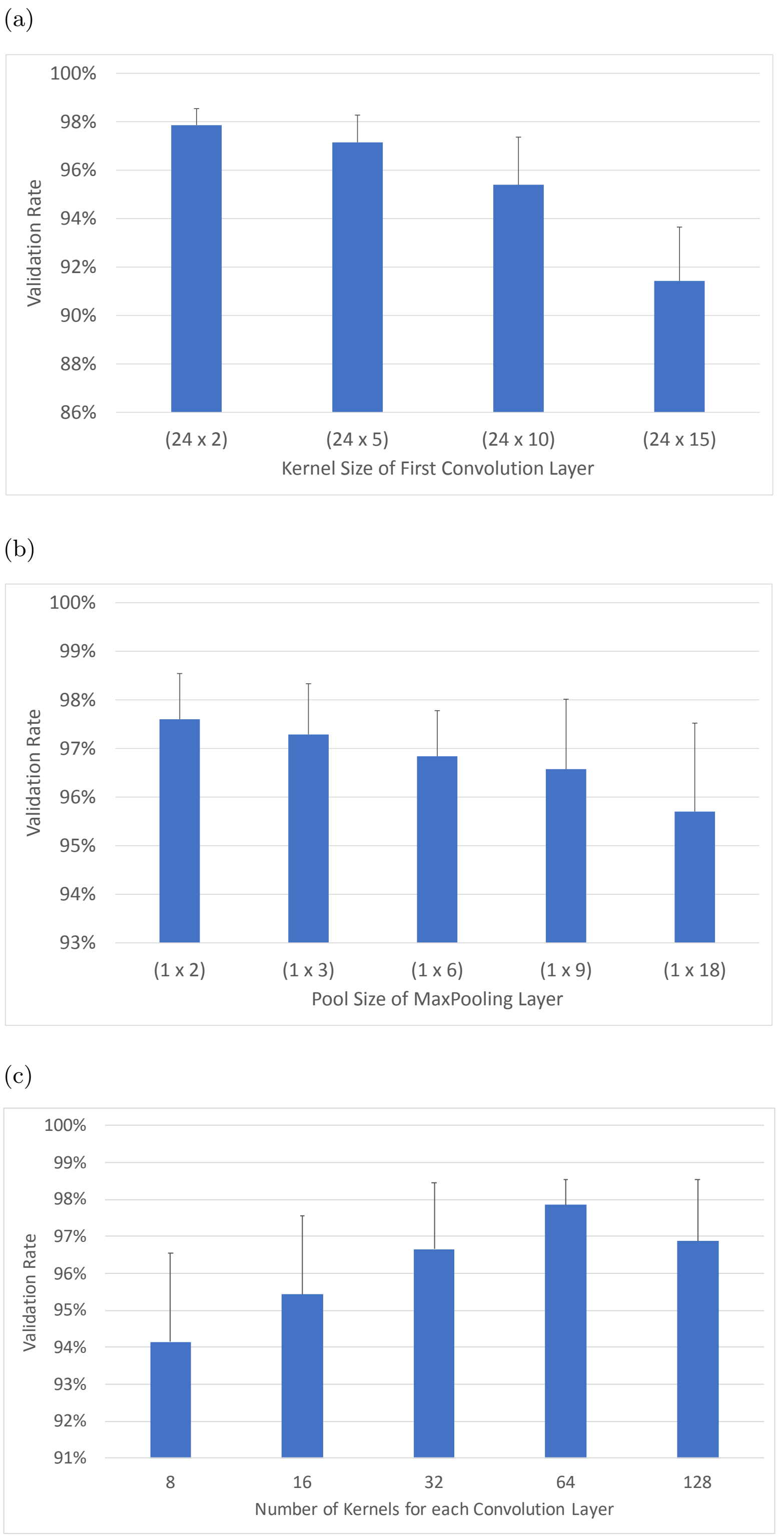

Figure S2: Sensitivity analysis on the size of convolutional layers, pooling layer, and the number of kernels, of convolutional layers. Figure S2a shows the validation rate as a function of the size of the kernels of the first convolutional layer. We observe that the larger the size of kernels , the smaller the validation rate. Since varying the size of the kernels the second convolutional layer has the same similar pattern as varying the size of the kernels of the first convolutional layer, we omit to show the results. Figure S2b shows the validation rate as a function of the size of pooling window of the pooling layer. We observe that the larger the size of window, the smaller the validation rate. Figure S2c shows the validation rate as a function of the number of the kernels of each convolutional layers. We observe that the validation rate is continuously increased until the number of kernels is 64. Beyond that, we find the lower validation rate when used 128 kernels for each convolutional layer.
